# Automated Optical 3d Scanner to Assess Postoperative Edema Following Forefoot Surgery

**DOI:** 10.1002/jfa2.70119

**Published:** 2025-12-29

**Authors:** Julien Beldame, Alessandro Civinini, Marie Aude Munoz, Riccardo Sacco, Amine Hamza, Matthieu Lalevee, Marie‐Anne Melone

**Affiliations:** ^1^ Institut de la Cheville et du Pied Amiens Amiens France; ^2^ Institut de la Cheville et du Pied Paris Paris France; ^3^ Normandie University, UNIROUEN, CETAPS UR3832, Groupe d'Etude Normand Interdisciplinaire de l'Appareil Locomoteur (GENIAL) Rouen France; ^4^ Department of Orthopedics CHU Rouen Rouen France; ^5^ Orthopedic Unit, Department of Health Sciences University of Florence C.T.O. Largo Palagi Firenze Italy; ^6^ Polyclinique Saint‐Roch Montpellier France; ^7^ Department of Pulmonary Thoracic Oncology and Respiratory Intensive Care Rouen France

**Keywords:** 3d scanner, bunion, edema, foot and ankle, forefoot

## Abstract

**Background:**

Postoperative edema is a common and often underestimated consequence of forefoot surgery, potentially affecting wound healing, patient comfort, and recovery time. Traditional measurement techniques, such as water displacement and circumferential taping, have limitations in the immediate postoperative period due to infection risk and operator dependence. This study aimed to quantify and monitor with a 3D optical scanner the postoperative edema following forefoot surgery and to identify risk factors associated with increased edema volume.

**Methods:**

In this prospective, single‐center observational study, 108 patients undergoing forefoot surgery were evaluated using the UPOD‐S 3D optical scanner. Each patient underwent serial volume assessments of both feet at baseline (Day 0, preoperatively), and postoperatively at Days 8, 21, 45, and 3 months. Patients were classified into three groups based on the type of surgery: isolated first ray procedures, combined first ray and lesser rays procedures, and isolated lesser ray procedures. A mixed‐effects multivariate model was used to evaluate the impact of sex, age, BMI, surgical type, and postoperative footwear on volume changes.

**Results:**

The operated foot showed a significant increase in volume compared to baseline at all postoperative timepoints (*p* < 0.001), peaking at Day 21 (+78 cm^3^; +9%). Also, when compared to Day 8 postoperative, foot volume increased by 25 cm^3^ at Day 21 (*p* = 0.008) and decreased by 27 cm^3^ at 3 months (*p* = 0.003). Male sex (+169 cm^3^, *p* < 0.001) and elevated BMI (BMI 26–30: +59 cm^3^; BMI > 30; +109 cm^3^; *p* < 0.01) were independent predictors of increased edema. Surgery involving combined hallux and lesser rays led to greater volume increases (*p* > 0.001). Age and type of postoperative footwear had no significant impact.

**Conclusion:**

We monitored forefoot surgery postoperative edema using 3D optical scanning. Male sex, elevated BMI, and multiple forefoot procedures were found to be key risk factors for prolonged or increased edema, which typically peaked at Day 21 and decreased thereafter in our study.

## 1 Introduction

Edema is characterized by the excessive accumulation of fluid in interstitial tissues. This buildup arises primarily from an imbalance between fluid filtration into tissues via capillaries and its removal through venous and lymphatic drainage. Postoperative reductions in venous return further exacerbate this accumulation [1]. Additionally, orthostatic pressure worsens limb edema, contributing to increased pain, delayed wound healing, and prolonged recovery times.

Measuring postoperative edema remains challenging due to constraints related to wound healing. Techniques relying on water displacement, though historically precise, are often contraindicated in postoperative settings due to the risk of infection or disruption of the surgical site [2, 3]. Circumferential measurements using tape are commonly used in clinical practice for their simplicity, but they are operator‐dependent and prone to variability [4, 5]. Volumetric assessments using optoelectronic techniques and bioimpedance spectroscopy offer reliable and accurate alternatives, but require specialized equipment and controlled environments, limiting their widespread use [6, 7]. Geometric or algorithmic methods (such as the figure‐of‐eight or circumference‐based algorithms) offer acceptable accuracy and practicality, yet remain highly sensitive to measurement technique [2, 3, 8].

Although various strategies such as limb elevation, compression therapy, cryotherapy, and early mobilization are available, since accurately assessing and monitoring postoperative edema remains challenging, this could lead to delay both in diagnosis and management of this condition.

In recent years, 3D scanning techniques have emerged as a promising solution, offering highly accurate and reproducible measurements without the need for contact or immersion, thus overcoming typical postoperative limitations and enabling detailed volume analysis [9].

Multiple studies have supported the precision and clinical applicability of these devices. Lee et al. showed that 3D foot scanning provides more accurate anthropometric measurements than conventional methods [9]. The UPOD‐S optical scanner was validated in previous studies, confirming its reliability in capturing a wide range of foot and ankle parameters [10]. Similarly, it was demonstrated that portable 3D scanners offer volumetric measurements comparable to those obtained by water displacement, the historical gold standard [11].

These findings support the integration of 3D optical scanning as a reliable, operator‐independent tool for edema assessment in the postoperative setting, particularly following forefoot surgery.

The aim of this study was to quantify and monitor changes in foot and ankle edema over time in patients undergoing forefoot surgery. A secondary objective was to determine which types of forefoot surgical procedures are associated with the highest risk of postoperative edema, and to identify demographic risk factors contributing to its development.

## 2 Materials and Methods

This study is a single‐center, observational, single‐operator study (JB, senior foot and ankle surgeon). This non‐interventional study received approval from the GHT Grand Paris Nord Est ethics committee on September 29, 2021 (reference number 21.01741.000023, registration number RCB 2021‐A01802.39).

Inclusion criteria consisted of adult patients undergoing surgery involving the hallux and/or lesser rays. Exclusion criteria included patients undergoing other types of forefoot surgery, or individuals unable to comply with the follow‐up protocol due to psychological, social, familial, geographical, or language‐related limitations.

A total of 108 patients were included in the study: 14 men and 94 women, with a mean age of 64 years. Surgery was performed on the right foot in 49% of cases and on the left in 51%.

Patients were classified into three groups based on the type of surgical procedure:

- Group 1: 47 forefeet operated exclusively on the first ray.
- Group 2: 46 forefeet operated on both the first ray and the lesser rays.
- Group 3: 15 forefeet operated exclusively on the lesser rays (with no first ray intervention).

Procedures involving the first ray included minimally invasive Chevron osteotomy of the first metatarsal (M1) combined with an Akin osteotomy (varisation of the proximal phalanx) [12], both fixed by a screw, or open metatarsophalangeal (MTP) joint arthrodesis using compression plates and screws. Surgery on the lesser rays was performed percutaneously and without fixation (distal metatarsal minimally invasive osteotomies, DMMO) [13].

Postoperative management followed routine clinical protocols, with bandaging maintained until Day 21, followed by inter‐toe orthoplasty until Day 31.

Postoperative footwear included either the HALTEN shoe (Podonov, France) used in 60 cases (56%), or the PODALUX shoe (DJO Global, United States) in 48 cases (44%). Footwear was maintained until Day 31, after which patients transitioned to wide, comfortable shoes (e.g., sneakers). Patients were prescribed self‐directed rehabilitation, with written and video instructions, physiotherapy until Day 21, and class II compression stockings. The total follow‐up duration was 3 months.

The measurement tool was the UPOD‐S 3D Laser Full‐Foot Scanner, a portable optical scanning device (East Lake, Wuhan City, Hubei Province, China 430,075) specifically designed for detailed foot and ankle analysis. Weighing only 13 kg and measuring 27 × 52 × 22 cm, it generates a precise 3D model of the foot and ankle up to 11.5 cm in height above the plantar surface, with a manufacturer‐reported accuracy of 0.5 mm. This device had been previously validated against conventional techniques and shown to provide reliable and accurate anthropometric data [9, 11]. The scanner automatically generates a PDF report containing approximately 40 anthropometric parameters (length, width, height, and circumferences), identified via the integrated UPOD 3D Full Foot Scan software [14]. In addition, we confirmed the reproducibility of its volume measurements using Blender software (Blender Foundation, Amsterdam, Netherlands), further supporting its clinical applicability [11].

**FIGURE 1:**
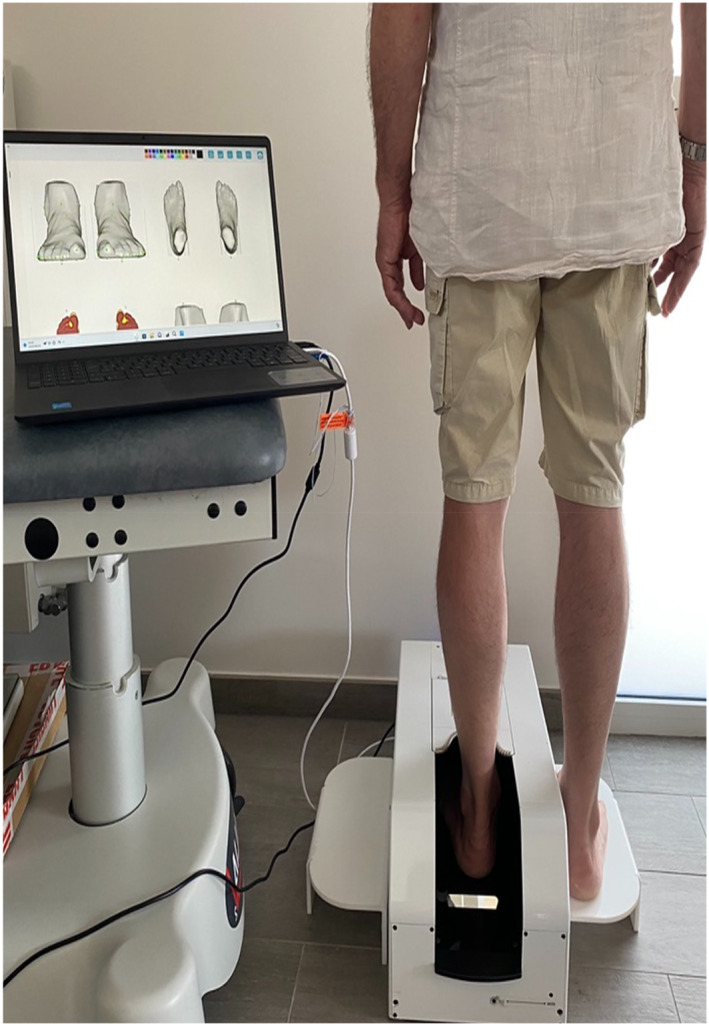
Scanned foot placed inside the scanner on a support surface, whereas the contralateral foot resting at the same level outside the scanner.

As previously described, patients were scanned in a standing upright position [11]. Each foot was scanned individually, whereas the subject maintained an even, balanced stance (bipedal support). The foot to be scanned was placed inside the scanner on a support surface, whereas the contralateral foot rested at the same level outside the scanner (Figure 1). Scans were conducted preoperatively at Day 0, and on postoperative Days 8, 21, 45, and at 3 months.

For the purposes of this study, we measured the total weight‐bearing volume of the foot and ankle up to a height of 9.5 cm above the plantar surface. This measurement was performed systematically on both feet at every follow‐up visit.

### 2.1 Statistical Analysis

Quantitative variables were described using median and interquartile range, and categorical variables by counts and percentages. A multivariate analysis was conducted to identify factors associated with variations in foot volume. A mixed‐effects model was employed, considering patient characteristics (age, sex, BMI), type of surgery and postoperative management (type of postoperative footwear). All analyses were performed using statistical software, and results were reported with medians and interquartile ranges for quantitative variables and frequencies for categorical variables. Statistical analyses were performed using software R statistics 4.4.1 and a significance level (alpha) was set at 0.05.

For each operated foot, the non‐operated contralateral foot was used as a control.

## 3 Results

Preoperative data showed that initial foot volumes were not significantly different between the foot scheduled for surgery and the control foot (859 cm^3^ ± 131 vs. 868 cm^3^ ± 132, respectively). During follow‐up, the volume of the control foot remained stable at Days 8, 21, 45, and at month 3 compared to baseline values (Table 1).

**TABLE 1:** Comparison of foot volume (cm^3^) between operated and control foot.

	Control foot *n* = 108	Operated foot *n* = 108	*p*
	Volume (cm^3^) ± SD	Volume (cm^3^) ± SD	
Day 0	859 ± 131	868 ± 132	*p* > 0.05
Day 8	859 ± 133	921 ± 142	*p* < 0.001
Day 21	860 ± 125	951 ± 93	*p* < 0.001
Day 45	859 ± 130	913 ± 142	*p* < 0.001
Month 3	858 ± 130	895 ± 140	*p* < 0.001

Abbreviation: SD, standard deviation.

**FIGURE 2:**
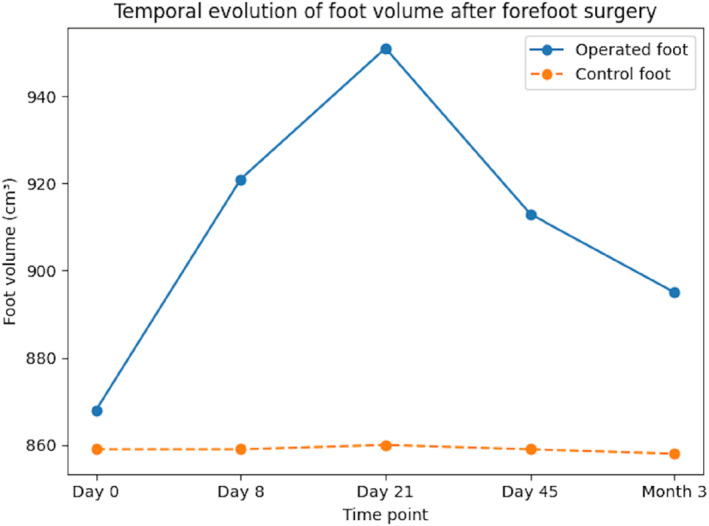
Temporal evolution of foot volume following forefoot surgery.

In contrast, the operated foot, when compared to baseline (Day 0), showed a significant volume increase at all postoperative timepoints: +54 cm^3^ at Day 8 (a 6% increase), +78 cm^3^ at Day 21 (the peak, corresponding to a 9% increase), +42 cm^3^ at Day 45 (a 4.9% increase), and +27 cm^3^ at month 3 (a 3.1% increase) (all *p* < 0.001) (Table 2) (Figure 2).

**TABLE 2:** Delta change in foot volume (cm^3^) compared to baseline (before surgery) in operated foot and control foot.

	Delta change in operated foot volume (cm^3^)			Delta change in control foot volume (cm^3^)		
	Delta	95% CI	*p* value	Delta	95% CI	*p* value
Day 0	—	—		—	—	
Day 8	+54	38, 71	< 0.001	+0.49	−4.1, 5.1	0.8
Day 21	+78	62, 94	< 0.001	−0.27	−4.8, 4.2	> 0.9
Day 45	+42	26, 59	< 0.001	−5.0	−9.5, −0.4	0.06
Month 3	+27	11, 43	< 0.001	−2.0	−6.4, 2.4	0.4

Abbreviation: CI, confidence interval.

When comparing operated foot volume to the volume recorded at Day 8, in order to obtain a comparison with the initial surgical edema, we found that volume peaked at Day 21 with a mean increase of +25 cm^3^ [95% CI 6.6, 43], corresponding to a 3% increase compared to Day 8 (*p* = 0.008). Volume returned to the Day 8 level at Day 45, and significantly decreased at month 3 compared to Day 8 (−27 cm^3^ [95% CI −45, −9.2], a 3% decrease, *p* = 0.003) (Table 3).

**TABLE 3:** Delta change in foot volume (cm^3^) compared to Day 8 in operated foot.

	Delta change in foot volume (cm^3^)	95% CI	*p* value
Day 8	—	—	
Day 21	25	6.6, 43	0.008
Day 45	−11	−29, 8.0	0.3
Month 3	−27	−45, −9.2	0.003

Abbreviation: CI, confidence interval.

In multivariate analysis, male sex and BMI ≥ 26 kg/m^2^ were identified as independent risk factors for postoperative foot volume increase. Mean postoperative foot volume was significantly higher in men compared to women (+169 cm^3^ [95% CI 112, 226] from Day 0 to month 3, *p* < 0.001). No significant association was found between age and volume variation.

Compared to patients with a normal BMI, those classified as overweight (25 < BMI < 30) showed an additional volume increase of 59 cm^3^ [95% CI 16, 102], corresponding to a 6.8% increase in foot volume compared to the average in patients with normal BMI (*p* = 0.009). Patients with a BMI > 30 had an additional increase of 109 cm^3^ [95% CI 52, 166], corresponding to a 12.7% increase (*p* < 0.001) (Table 4).

**TABLE 4:** Risk factors of postoperative increased foot volume (cm^3^) according to age, sex and BMI, type of surgery and type of postoperative footwear.

	Beta	95% CI	*p* value
Sex			
Female	—	—	
Male	169	112, 226	< 0.001
Age (year)			
< 50	—	—	
51–65	16	−41, 74	0.6
66–75	19	−44, 82	0.6
> 75	−68	−148, 12	0.1
BMI (kg/m^2^)			
< 25	—	—	
26–30	59	16, 102	0.009
> 30	109	52, 166	< 0.001
Postoperative footwear			
Halten	—	—	
Podalux	22	−18, 61	0.3
Type of surgery			
Exclusively on the first ray	—	—	
Both the first ray and the lesser rays	8.4	−34, 51	0.7
Exclusively on the lesser rays	−3.4	−62, 56	> 0.9

Abbreviations: BMI, body mass index; CI, confidence interval.

Patients undergoing surgery on both the first ray and the lesser rays experienced a significantly greater volume increase at Day 8, Day 45, and month 3 compared to those who underwent surgery on the first ray or the lesser rays alone (Table 5).

**TABLE 5:** Comparison of percentage volume variation (compared to baseline volume, Day 0) according to each type of surgery.

Phase	Group 1 *N* = 47^a^	Group 2 *N* = 46^a^	Group 3 *N* = 14^a^	*p* value
Day 8	5.1 +/−3.8	8.8 +/−6.0	4.7 +/−4.9	0.004
Day 21	7.2 +/−4.0	9.2 +/−3.7	7.1 +/−5.2	0.6
Day 45	3.0 +/−4.0	7.3 +/−4.8	2.4 +/−3.4	< 0.001
Month 3	1.7 +/−4.2	4.5 +/−4.5	2.7 +/−5.1	0.018

^a^ Mean +/− standard deviation.

Finally, no postoperative complications were reported in the cohort, including infections, embolisms, hardware displacement, or reoperations.

## 4 Discussion

Our study quantitatively assessed postoperative edema following forefoot surgery using a 3D optical scanner. The combination of a large patient sample and a validated measurement tool allowed us to characterize the specific kinetics of edema in this type of surgery, which was found to peak at Day 21.

The observed edema followed a distinctive pattern, which could be unique to forefoot surgery: volume increased between Day 8 and Day 21, then gradually decreased by Day 45 and continued to decline through month 3. This contrasts with the typical timeline of postoperative edema observed in other orthopedic procedures, which, according to Kullenberg et al., generally peaks between Days 4 and 7 following surgery, as demonstrated in their prospective study of 86 patients undergoing total knee arthroplasty [15]. This delayed peak may be explained by the compressive effect of specific postoperative bandaging used in forefoot surgery. Our findings are consistent with those of Lehnert et al., who reported that foam‐based postoperative dressings provided both compression and immobilization, reducing inflammation, edema, and bruising [16]. However, their protocol included foam only during the first five postoperative days, though they suggested prolonged use could be beneficial.

Although no study seems to directly evaluate the specific link of BMI and increased edema risk in forefoot surgery, our multivariate analysis, which identified BMI as independent risk factors for increased postoperative volume seems to be in accordance with studies analyzing edema incidence and its pathophysiological relationship with obesity [17, 18].

BMI emerged indeed as the most influential risk factor for edema, with increases of + 59 cm^3^ (6.8%) in patients with BMI 26–30 and + 109 cm^3^ (12.7%) in patients with BMI > 30, compared to individuals with normal BMI. This may partially explain the delayed wound healing and higher infection rates observed in patients with elevated BMI, beyond the sole contribution of obesity‐related inflammatory mechanisms [19].

Male sex was also a significant risk factor, aligning with previous research: McWhorter et al. [20] reported greater edema in men following physical activity, whereas González‐Martín et al. [21] noted a higher incidence of intraosseous edema in male patients. In contrast, age was not associated with increased postoperative edema in our study.

These results suggest that postoperative edema is influenced primarily by phenotypic factors such as sex and BMI, and by the extent of surgical intervention, rather than by patient age or type of postoperative footwear.

Most orthopedic studies on postoperative edema have focused on treatment strategies, particularly compression therapies in the context of ankle fractures. Although pneumatic compression has shown some benefit, its effectiveness is generally assessed using circumferential measurements, such as ankle circumference or the figure‐of‐eight method, which lack volumetric precision [22, 23, 24]. As a result, precise and reliable volumetric assessment of edema is still largely missing from the literature [25]. This lack of data is likely related to the methodological challenges of measuring edema volume accurately [26, 27].

Only two prior studies have attempted to quantify postoperative edema using the gold standard of water displacement. Thordarson et al. studied 30 patients preoperatively but could not conduct immediate postoperative measurements [28]. Myerson et al. assessed postoperative edema only after wound healing, once patients could safely immerse the foot in water [29]. Similarly, Thordarson et al. delayed measurements to avoid wound complications, typically performing them around 3 weeks postoperatively [28]. Thus, the early postoperative period, when edema is maximal and complications are most likely, remained unquantified [30, 31]. Moreover, due to pain and incomplete bone healing, measurements were conducted under partial weight‐bearing, introducing bias.

Man and Morrissey assessed ankle edema in 36 patients after sprain using water displacement, but their small sample size limited the statistical power of their results, both in quantifying edema and identifying associated factors [32].

This literature highlights the need for reliable, non‐invasive tools capable of accurately assessing postoperative edema, especially during the early stages. In this context, 3D optical scanning offers a valuable solution, as it enables precise volumetric measurements without requiring contact or immersion, thereby overcoming many of the methodological limitations described [10, 11].

This study presents some limitations, notably, despite the relatively large cohort in our study, it was conducted by a single surgeon, which may have introduced selection bias, particularly in the distribution of surgical groups. For instance, only 15 patients (13%) underwent isolated lesser rays surgery, possibly limiting statistical comparisons across all three surgical subgroups. Furthermore, the 3‐month follow‐up period did not allow us to determine the complete timeline for edema resolution or stabilization.

## 5 Conclusion

In our study involving 3D optical scanner assessment, postoperative edema following forefoot surgery peaked at Day 21 (+78 cm^3^, corresponding to a 9% increase compared to Day 0 preoperatively), started to improve by Day 45 and further decreased at 3 months.

BMI emerged as the main risk factor, with increases of +6.8% for BMI > 25 and + 12.7% for BMI > 30. Male sex was also associated with a significant increase in foot volume (+169 cm^3^ compared to females). Furthermore, surgeries involving a combination of first ray and lesser rays procedures resulted in greater edema than isolated interventions.

## Author Contributions

**Julien Beldame:** conceptualization, methodology, investigation, data curation, writing – original draft, supervision. **Alessandro Civinini:** conceptualization, methodology, formal analysis, writing – original draft, writing – review and editing. **Marie‐Aude Munoz:** investigation, data curation, writing – review and editing. **Riccardo Sacco:** methodology, software, validation, writing – review and editing. **Amine Hamza:** formal analysis, data curation, writing – review and editing. **Matthieu Lalevée:** conceptualization, methodology, supervision, writing – review and editing. **Marie‐Anne Melone:** investigation, resources, writing – review and editing.

## Funding

The authors have nothing to report.

## Conflicts of Interest

The authors declare no conflicts of interest.

## Data Availability

The data that support the findings of this study are available from the corresponding author upon reasonable request.
